# Nasoseptal chondroprogenitors isolated through fibronectin-adherence confer no biological advantage for cartilage tissue engineering compared to nasoseptal chondrocytes

**DOI:** 10.3389/fbioe.2024.1421111

**Published:** 2024-09-26

**Authors:** Thomas H. Jovic, Emman J. Thomson, Nick Jones, Catherine A. Thornton, Shareen H. Doak, Iain S. Whitaker

**Affiliations:** ^1^ Reconstructive Surgery and Regenerative Medicine Research Centre, Institute of Life Sciences, Swansea University, Swansea, United Kingdom; ^2^ Welsh Centre for Burns and Plastic Surgery, Morriston Hospital, Swansea, United Kingdom; ^3^ Institute of Life Sciences, Swansea University Medical School, Swansea University, Swansea, United Kingdom

**Keywords:** chondroprogenitor, cartilage, tissue engineering, fibronectin, stem cell

## Abstract

**Introduction:**

The ability to bioprint facial cartilages could revolutionise reconstructive surgery, but identifying the optimum cell source remains one of the great challenges of tissue engineering. Tissue specific stem cells: chondroprogenitors, have been extracted previously using preferential adhesion to fibronectin based on the expression of CD49e: a perceived chondroprogenitor stem cell marker present on <1% of cartilage cells. This study sought to determine whether these fibronectin-adherent chondroprogenitor cells could be exploited for cartilage tissue engineering applications in isolation, or combined with differentiated chondrocytes.

**Methods:**

Nasoseptal cartilage samples from 20 patients (10 male, 10 female) were digested to liberate cartilage-derived cells (CDCs) from extracellular matrix. Total cell number was counted using the Trypan Blue exclusion assay and added to fibronectin coated plates for 20 min, to determine the proportion of fibronectin-adherent (FAC) and non-adherent cells (NFACs). All populations underwent flow cytometry to detect mesenchymal stem/progenitor cell markers and were cultured in osteogenic, chondrogenic and adipogenic media to determine trilineage differentiation potential. Cell adherence and growth kinetics of the different populations were compared using iCELLigence growth assays. Chondrogenic gene expression was assessed using RT-qPCR for Type 2 collagen, aggrecan and SOX9 genes. Varying proportions of NFAC and FACs were cultured in alginate beads to assess tissue engineering potential.

**Results:**

52.6% of cells were fibronectin adherent in males and 57.7% in females, yet on flow cytometrical analysis, only 0.19% of cells expressed CD49e. Moreover, all cells (CDC, FAC and NFACs) demonstrated an affinity for trilineage differentiation by first passage and the expression of stem/progenitor cell markers increased significantly from digest to first passage (CD29, 44, 49e, 73 and 90, p < 0.0001). No significant differences were seen in adhesion or growth rates. Collagen and aggrecan gene expression was higher in FACs than CDCs (2-fold higher, p = 0.008 and 0.012 respectively), but no differences in chondrogenic potential were seen in any cell mixtures in 3D culture models.

**Conclusion:**

The fibronectin adhesion assay does not appear to reliably isolate a chondroprogenitor cell population from nasoseptal cartilage, and these cells confer no advantageous properties for cartilage tissue engineering. Refinement of cell isolation methods and chondroprogenitor markers is warranted for future nasoseptal cartilage tissue engineering efforts.

## 1 Introduction

Cartilage is a tissue of very limited regenerative capacity, owing to its avascularity, as evidenced by degenerating diseases of cartilage such as osteoarthritis (Tuan et al., 2013). As such, there has been interest in identifying cells capable of cartilage regeneration: encompassing tissue specific stem/progenitor cells, mesenchymal stem/progenitor cells and induced pluripotent stem cells (Jessop et al., 2019). In light of the limitations of non-cartilaginous cell sources, the pursuit of a chondroprogenitor cell population within cartilage has been a highly sought solution, offering the potential to regenerate *de novo* cartilage tissue through tissue engineering (Williams et al., 2010a; Jessop et al., 2016; Jessop et al., 2019).

The success of tissue engineering is determined by the combination of cells, scaffold and culture environment (Song et al., 2004). With regard to optimal cell selection for generating *de novo* cartilage, competing strategies have been employed to generate cartilage *in vitro* (Jessop et al., 2016). Mesenchymal stem/progenitor cells such as bone marrow derived stem cells and adipose derived stem cells have gained traction owing to their multipotency and accessibility for harvest (Raghunath et al., 2005; Xie et al., 2012; Veronesi et al., 2014; Yamasaki et al., 2014; Rosadi et al., 2019). However, stem/progenitor cells acquired from unrelated tissue sources have proven problematic in clinical translation, resulting in poor quality cartilage formation prone to degradation, calcification, and mechanical instability (Cao et al., 1997; Kusuhara et al., 2009; Bichara et al., 2012). The use of induced pluripotent stem cells has been explored for tissue engineering, but concerns remain about the efficiency of redifferentiation and the potential for neoplastic development owing to the genetic reprogramming of pluripotency (Medvedev et al., 2010; Yamashita et al., 2013).

As such, tissue-specific stem/progenitor cells have been considered a panacea for tissue engineering and bioprinting biomimetic tissue (Jessop et al., 2019). In 2004, a small population of cells demonstrating high colony forming efficiency, adhesion to fibronectin and Notch1 expression were identified, and hypothesized to represent a novel chondroprogenitor population (Dowthwaite et al., 2004). This proposed chondroprogenitor population is believed to comprise a very small proportion of the total cell population, comprising approximately 0.7% of cells in articular cartilage (Williams et al., 2010a). It has been proposed that these chondroprogenitor cells can be isolated through their capacity to bind fibronectin: a glycoprotein involved in organising extracellular matrix components such as collagen and fibrin (Grogan et al., 2007; Williams et al., 2010a) and mediating cell adhesion (Pankov and Yamada, 2002). This isolation technique is based on their high levels of cell surface adhesion proteins, such as α5β1 integrin (a dimer of CD49e-CD29), of which CD49e has been detected on a small number of the cartilage cells believed to represent progenitor cells (Williams et al., 2010b; Kachroo et al., 2020).

Reports of isolating cartilage specific stem/progenitor cells have now been described from cartilage tissue of the intervertebral disks, auricle, nasoseptum, trachea and costal cartilages (Jessop et al., 2019). Believed to have a key role in mediating tissue homeostasis, this elusive stem/progenitor cell population has been speculated to possess the properties of a mesenchymal stem/progenitor cell as defined by the International Society of Cellular Therapy (Dominici et al., 2006): Plastic adherence in standard culture conditions, expression of key cell markers such as CD73 and CD90 with an absence of markers such as CD45 and CD34, plus the ability to undergo trilineage differentiation into osteoblasts, adipocytes and chondrocytes *in vitro*.

A number of different techniques have been described regarding chondroprogenitor isolation, with some studies adopting flow cytometric cell sorting based on key surface markers (Hattori et al., 2007; Grogan et al., 2009), some using fibronectin adherence (Dowthwaite et al., 2004; Anderson et al., 2018; Jessop et al., 2020) and others identifying migratory cells from perichondrial tissue (Koelling et al., 2009; Seol et al., 2012). Irrespective of the methods of isolation, most of the chondroprogenitor cells isolated from cartilage tissue express comparable cell surface markers: CD90, 105, 44, 166, 73 and 29 plus an absence of CD34 and 45 expression (Jessop et al., 2019). The exception to this pattern was found to be chondroprogenitor cells isolated from intervertebral disks which arise from different embryological origins: the primitive notochord (Risbud and Shapiro, 2011). Additionally, most of the chondroprogenitor cells isolated demonstrate multi-lineage potential, in particular osteogenic but also chondrogenic and to a lesser reported degree, adipogenic lineages (Jessop et al., 2019). In spite of these findings, chondroprogenitor cells remain a controversial topic, with recent studies disputing the validity of fibronectin adherence and the role of CD49e in serving as a marker of the progenitor population in articular cartilage (Kachroo et al., 2020; Vinod et al., 2020). However, such validation has not been performed in nasoseptal chondrocytes, particularly with a view to assessing the potential for different combinations of nasoseptal chondrocyte populations for tissue engineering applications.

This study therefore aimed to validate the reliability of the fibronectin adhesion assay in isolating chondroprogenitor cells from nasoseptal cartilage and to evaluate whether these cells confer any beneficial properties for tissue engineering applications, both in isolation and in combination with non-progenitor cells.

## 2 Methods

### 2.1 Isolation of cartilage derived cells

Human nasoseptal chondrocytes were isolated from nasoseptal cartilage remnants following septorhinoplasty procedures from ten male and ten female patients through digestion in pronase for 40 min (2 mg/mL, Roche, Basel, Switzerland) and collagenase for 16 h (2.4 mg/mL, Sigma-Aldrich, MO, United States) at 37°C as described previously (Jessop et al., 2020). The mixed population of cells acquired from this enzymatic digest should contain a mixture of chondroprogenitor cells and chondrocytes referred to hereafter as Cartilage Derived Cells (CDCs). Isolation of a suspected chondroprogenitor cell population has been described previously using a fibronectin adhesion assay. In brief, culture vessels are coated with a 10 mg/mL fibronectin (Sigma Aldrich), solution made in Dulbecco’s Phosphate Buffered Saline (pH 7.4, Thermofisher Scientific) containing 0.5 mM magnesium chloride and 0.9 mM calcium chloride, at least 24 h prior to the addition of cells. The CDC mixture, suspended in media, is added to the fibronectin-coated plate immediately post-digest and the chondroprogenitor cells are believed to adhere to the cells within 20 min (Jones and Watt, 1993; Williams et al., 2010b). The cells that adhere to the fibronectin within this period are referred to in this study as fibronectin-adherent cells (FACs): the presumed chondroprogenitor population. The remaining media contains CDCs that are not adherent to fibronectin within the 20-min period and are referred to as non-fibronectin adherent cells (NFACs). These cells should theoretically represent a chondrocyte population free from chondroprogenitors. All cells were cultured in Dulbecco’s Modified Eagle Medium with 10% fetal bovine serum, 0.1% glucose, 0.1% non-essential amino acids and 1% penicillin-streptomycin only (all acquired from Gibco, Thermofisher, MA, United States). Each of the cell populations were cultured in separate culture vessels post-separation, and allowed to proliferate up to 70% confluence before passaging.

### 2.2 Flow cytometric characterisation of cell populations

CDCs, FACs and NFACs were characterised using flow cytometry to examine expression of mesenchymal stem/progenitor cell and chondrogenic cell surface markers and to ensure that haematopoietic and non-mesenchymal lineage markers were not present on the cells. 0.1 × 10^6^ cells were added to flow tubes (Fisher Scientific) with CDCs taken immediately post-harvest (Passage 0, P0) and characterised for the surface expression of a panel of mesenchymal and chondrogenic stem/progenitor cell markers as previously optimised (Jessop et al., 2020). After splitting and separately culturing the cells to first passage (P1), the individual cell populations - CDCs, NFACs and FACs (Supplementary Figure S1)- were then characterised using the same flow cytometry panel. Cells from each population were transferred to 6 separate flow tubes to be processed as either unstained or stained with antibodies against various mesenchymal stem/progenitor cell, chondrogenic cell, haematopoietic and non-mesenchymal cell markers combined to minimise overlap in emission spectra as previously validated (Jessop et al., 2020) (all from Biolegend, CA, United States; Table 1). Specifically, cells were stained for CD29 (PE, mIgG1k, TS2/16, 303004), CD44 (PerCP, mIgG2b, IM7, 103035), CD56 (Brilliant Violet 605™, mIgG1k, 5.1H11, 362537) and CD73 (APC/Cyanine 7, mIgG1k, AD2, 344021); or CD90 (Brilliant Violet 510™, mIgG1k, 5E10, 328125); CD49e (FITC, mIgG2b, NKI-SAM-1, 328008); CD24 (Brilliant Violet 421™, mIgG2a, ML5, 311121), CD34 (APC/Cyanine7, mIgG1k, 581, 343513) and CD45 (Brilliant Violet 570™, mIgG1, HI30, 304033), or Stro-1 (Alexa Fluor^®^ 647, mIgMy, STRO-1, 340103). CD105 was not included owing to its lack of value in chondrogenic mesenchymal stem/progenitor cell characterization (Cleary et al., 2016). The cell pellet was then resuspended in flow assisted cell sorting (FACS) buffer consisting of 0.2% bovine serum albumin (Sigma Aldrich) and 0.05% sodium azide in phosphate buffered solution (Gibco, Thermofisher Scientific, MA, United States) with 5 μL of each fluorophore conjugated antibody added for 30 min protected from light on ice using a 1:20 dilution and processed as previously described (Jessop et al., 2020). The percentage of cells positive for each cell surface marker was ascertained relative to the unstained population (% cells positive) and the median fluorescence index (MFI) of the stained populations for each fluorophore was expressed relative to the MFI of unstained cells. Each experiment was performed in biological triplicates (three separate primary cell lines) for each passage and fluorophore specified.

**TABLE 1:** Cell surface markers, their associated fluorophores, detection channels and excitation and emission spectra.

CD marker	Other names	Fluorophore	Detection channel	Excitation wavelength (nm)	Emission wavelength (nm)
CD 29	Integrin β1, VLA-β, gpIIa	PE	PE	565	575
CD 44	Hermes, Pgp1, H-CAM, HUTCH	Per CP	APC	488	675
CD 56	NCAM, Leu-19, NKH1	BV 605	Q dot 605	405	605
CD 73	Ecto-5′-nucleotidase	APC/Cy 7	APC Cy7	650	774
CD 90	Thy-1	BV 510	Am Cy	405	510
CD49e	α5 integrin, VLA-5α	FITC	FITC	493	525
CD 24	HAS, Ly-52, Nectadrin	BV 421	Pacific Blue	405	421
CD 34	Gp105-120	APC/Cy7	APC Cy 7	650	774
CD 45	LCA, T200	BV 570	PE	405	570
Stro-1	-	AF 647	APC	650	668

### 2.3 Trilineage differentiation of cartilage cell populations

The potential of each cell population to differentiate into osteogenic, adipogenic and chondrogenic lineages was determined using StemPro trilineage differentiation kits (Thermofisher, MA, United States). In brief, cells from each of the CDC, FAC, and NFAC populations were seeded into 12 well plates (Thermofisher, MA, United States) and cultured for 24 h until adherent. Thereafter, the cells were cultured using chondrogenic, adipogenic or osteogenic media for up to 21 days. The cells were then fixed with 4% paraformaldehyde solution for 30 min and stained with lineage specific stains: Alizarin Red (bone), Alcian blue (cartilage) or Oil Red O stain (adipose) (Sigma Aldrich). All populations were also stained separately with haematoxylin and eosin to facilitate the characterisation of cell morphology. Duplicate wells were seeded using biological triplicates. Cell morphology was visualised using brightfield microscopy at 10× and 40× magnification using an Olympus CKX53 microscope, and images were captured using cellSens software (Standard Version, Olympus, Tokyo, Japan).

### 2.4 Cell ratio populations

Combined populations of FAC and NFAC cells were produced after first passage in the ratios outlined in Table 2.

**TABLE 2:** Cell ratios, their nomenclature and constituents.

Cell ratio	Proportion of NFAC cells (%)	Proportion of FAC cells (%)
CDC	Native proportion	Native proportion
100NFAC	100	0
80N:20F	80	20
60N:40F	60	40
40N:60F	40	60
20N:80F	20	80
100FAC	0	100

Mixed cell populations with different ratios of NFAC and FAC cells were achieved using cell counting with a trypan blue exclusion assay and mixing cells according to the proportions in Table 1. To assess tissue engineering potential, the cell suspensions were added to a 2.5% w/v alginate hydrogel (Merck, Darmstadt, Germany) at a density of 3 × 10^6^ cells per ml and dispersed into 100 µL beads (alginate beads) using a 1 mL syringe. The beads were crosslinked through the addition of 0.5 M CaCl_2_ for 5 min and cultured for up to 21 days with media changes (as per Section 2.1) every 3 days. Three technical repeats were performed for each biological repeat.

### 2.5 Chondrogenic gene expression

The cell populations from 3 biological repeats were harvested at first passage using TRIzol reagent (Thermofisher, MA, United States) and frozen at −80°C. The RNA from the lysate was extracted and processed as previously described (Jovic et al., 2023) the resultant mRNA was used to quantify gene expression using reverse transcription real-time quantitative polymerase chain reaction (RT-qPCR) as previously described (Jovic et al., 2023). The target genes used to assess for chondrogenic gene expression were Type 2 Collagen (COL2A1), Type 1 Collagen (COL1A1), aggrecan (ACAN) and SOX9 alongside the housekeeping genes RPL13A and tatabox protein (TBP), the primer sequences of which are available in Supplementary Table S1. Ct values were analysed for relative gene expression using the ddCt method. Four technical repeats were performed per biological sample.

### 2.6 Dimethylmethylene blue (DMMB) assay

A DMMB assay was conducted on 3 separate alginate beads per cell population using the same biological triplicates. Lysate was prepared by homogenisation with RIPA buffer (Sigma Aldrich) and protease inhibitor (Thermofisher Scientific, MA, United States) solution to extract intra- and extracellular extracellular matrix and diluted 1 in 50 with distilled water. Each lysate was reacted with 200 μL of DMMB solution and read at an absorbence of 525 nm along with a series of chondroitin sulphate (Sigma Aldrich) standards ranging from 0 to 50 μg/mL. This standard curve was used to calculate the glycosaminoglycan content of each sample. Absorbency values were corrected for media and alginate only control samples.

### 2.7 Cell adhesion and proliferation

The iCELLigence impedance-based assay (ACEA Biosciences, Agilent, CA, United States) was used to determine the adhesion and growth trajectories of the different unmixed and mixed cell populations over a 72-h period; 17,500 cells were seeded per well. A total of 6 biological repeats were assessed using duplicates per condition. During the first 2 h of the iCELLigence protocol, impedance readings were acquired every minute to record adhesion rates on to the polystyrene wells of E8 plates (ACEA Biosciences) as a marker of plastic adherence. Thereafter, hourly readings were acquired to capture changes in electrical impedance as a measure of cell proliferation. The data from the different populations were pooled across biological repeats and used to calculate growth curves, adhesion rates and population doubling times.

### 2.8 Histological analysis

Cells cultured in alginate beads were harvested at 21 days, immersed in paraformaldehyde and dehydrated with 30% sucrose solution. Dehydrated samples were then snap frozen in Optimum Cutting Compound (VWR, Pennsylvania, United States) and cryosectioned into 10 micron sections. 0.1% Alcian blue stain was applied to the slides for 30 min. Images were acquired at 40× magnification using an Olympus CKX53 microscope, and captured using cellSens software (Standard Version, Olympus, Tokyo, Japan).

### 2.9 Statistical analysis

Where possible, data sets were assessed for normality (Gaussian distribution) using an Anderson-Darling test to guide statistical test selection. Mann Whitney tests were used to compare FAC and NFAC ratios from nasoseptal digests from men and women. Relative gene expression data was compared using nested one way ANOVA analysis, with Tukey’s *post hoc* test, enabling comparison of gene expression differences whilst accommodating for variations in biological repeats. Flow cytometry data and iCELLigence data were compared using a one-way ANOVA with Tukey’s *post hoc* test. Twenty biological repeats were used for calculating native proportions of FAC and NFAC cells, 6 biological repeats were used for iCELLigence (in technical duplicates) and thereafter biological triplicates (performed in technical triplicates) were used for gene expression analysis and extracellular matrix (ECM) quantification.

## 3 Results

### 3.1 Native proportions of fibronectin adherent and non-fibronectin adherent cells in adult nasoseptal cartilage

Chondroprogenitor cells should constitute a relatively small proportion of the total nasoseptal chondrocyte cell population, and as such, the initial experiments sought to determine the proportion of CDCs immediately post-digest that were capable of fibronectin adherence within the 20-min interval (FACs). Thereafter, whether there were differences in the proportion of FACs in female and male patients was investigated in addition to any age-related changes.

**FIGURE 1:**
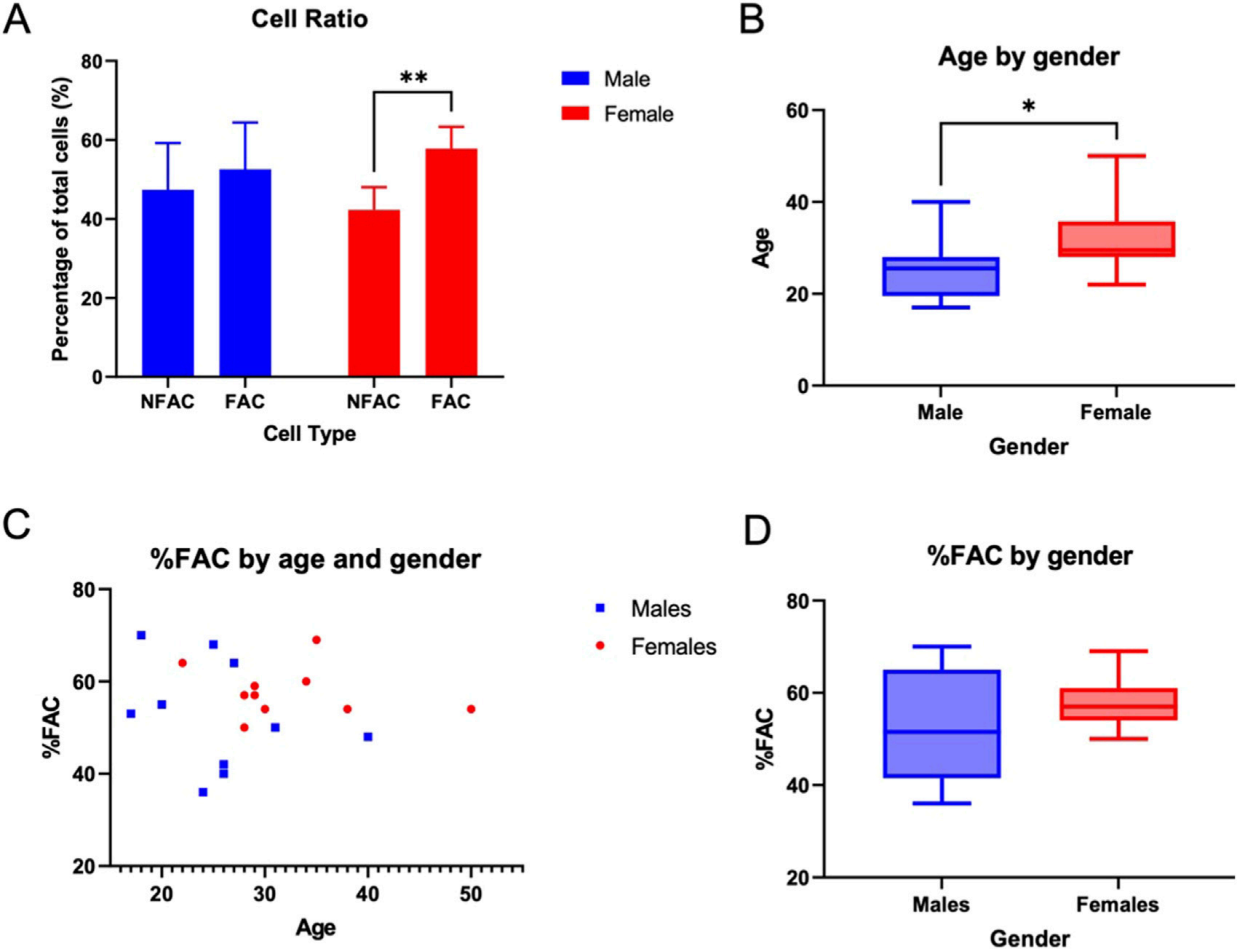
Proportion of fibronectin-non-adherent (NFAC) and fibronectin-adherent (FAC chondrocytes) from donor nasoseptal cartilage. **(A)** Mean values of proportion of NFAC and FAC cells from a set of 10 male and 10 female patients are presented with standard deviation (SD) error bars. There were no significant differences noted for the male donors. **(B)** Box and whisker plot of age (years) in male and female cohorts with boxes depicting IQR and median and whiskers depicting the range **(C)** Scatterplot of age in years (*x*-axis) compared to percentage of FAC cells (*y*-axis) of total cell numbers used to calculate correlation with Spearman’s rank correlation coefficient **(D)** Percentage of FAC cells (as a percentage of total cells) in male and female cohorts with boxes depicting IQR and median and whiskers depicting the range. * = p < 0.05; ** = p < 0.01.

The number of NFAC cells was lower than the number of FACs in both male (47.4%, n = 10) and female patients (42.3%, n = 10), though this was only a statistically significant difference in female patients (p = 0.001) Figure 1A.

Of the patients studied, the female cohort were significantly older than the male cohort studied (p = 0.02) with a median age of 25.5 years in the male cohort (IQR = 7, range 19) and 29.5 years in the female cohort (IQR 23.5, range 34) Figure 1B. The proportion of FACs did not appear to differ between male (median 51.5, IQR 18.25) and female (median 57, IQR 5.75) subjects, though a much broader range of FAC cells were observed in the male cohort (range 34) compared to females (range 19) Figures 1C, D. The trend in the proportion of FAC cells appeared to display minimal correlation with age in both male (y = −0.51x + 65.5; *R* ^2^ = 0.0834) and female (y = −0.14x+ 62.4; *R* ^2^ = 0.0382) patients using Spearman’s rank correlation coefficient. As such, age and gender were not associated significantly with the proportion of FACs in human nasoseptal cartilage, and the proportion of cells capable of fibronectin adherence using the fibronectin adhesion assay were in excess of 50% for both male and female patients. This value far surpasses the expected proportion of chondroprogenitor cells in a relatively senescent tissue such as cartilage if that definition is driven by fibronectin adherence capability.

### 3.2 Characterisation of cell populations using flow cytometry

The fibronectin adhesion assay is believed to selectively adhere chondroprogenitors on the basis of their expression of CD49e “the Fibronectin Receptor” (Williams et al., 2010b; Kachroo et al., 2020). As such, the number of CDCs expressing the CD49e receptor was determined immediately after digest and prior to fibronectin adhesion (CDC P0). If this assay were to successfully isolate CD49e positive cells, a value in excess of 50% would be anticipated, mirroring the cell counts of fibronectin adhesion.

**FIGURE 2:**
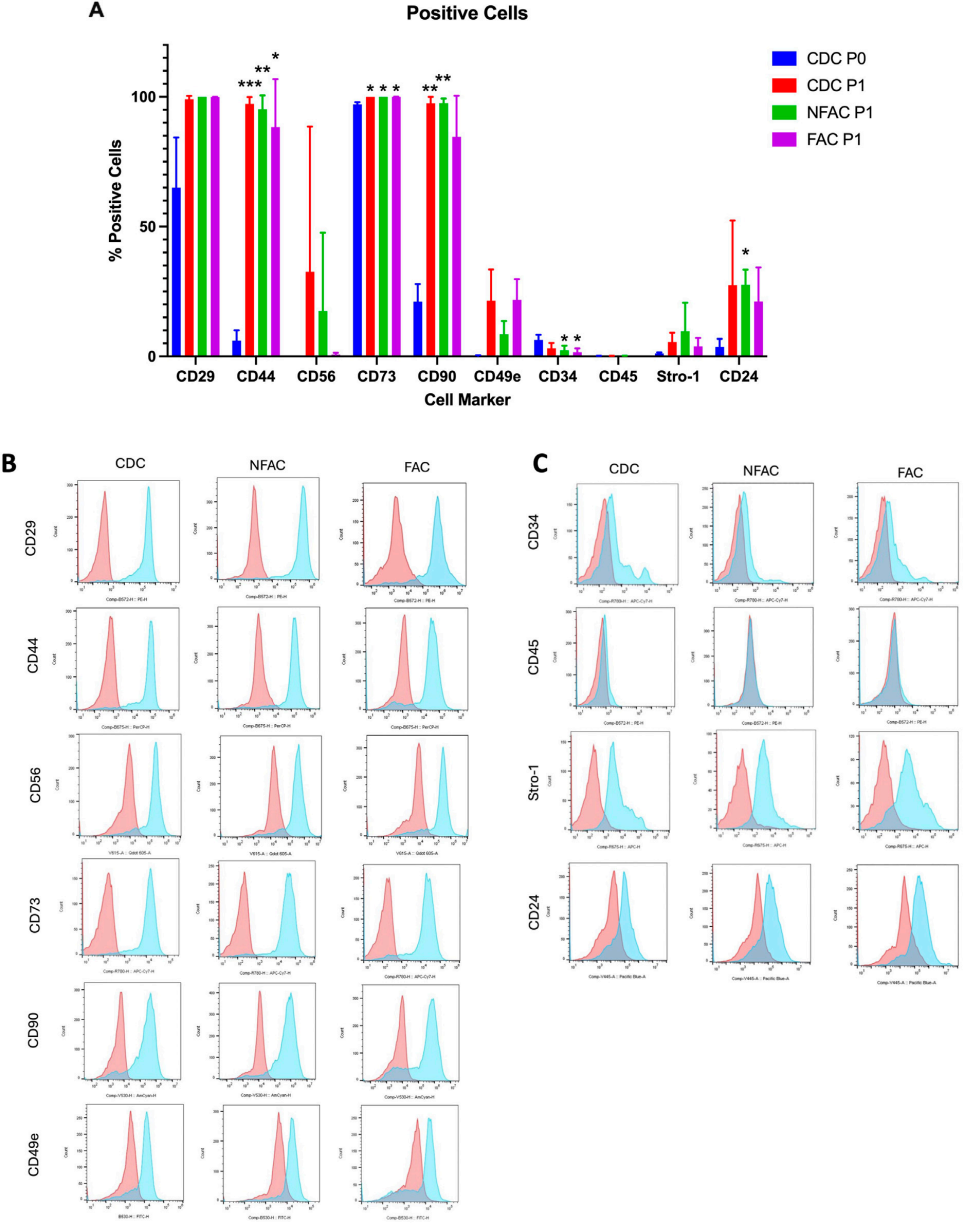
**(A)** Mean percentage of total cells expressing cell surface markers at P0 (blue) and P1 (CDC = red, NFAC = green, FAC = purple). The mean of a biological n = 3 is presented with error bars depicting standard deviation. **(B, C)** Flow cytometry curves from a representative sample are presented for each cell surface marker at Passage 1 with red curves indicating unstained and blue curves indicating stained populations. * = p < 0.05; ** = p < 0.01; *** = p < 0.001; **** = p < 0.0001.

The proportion of cells positive for each cell marker was expressed as a percentage of the total cell population (Figure 2A) and as a Median Fluorescence Index (MFI) as displayed in histogram format (Figure 2B). Signal:noise (S:N) ratios: a ratio of the median fluorescence index of the stained population relative to the unstained population to correct for artefactual fluorescence are available in Supplementary Figure S2. After 14 days of culture and first passage, the expression of cell surface markers was compared between the three different cell populations (CDC P1, NFAC P1, FAC P1) and the original cell population post digest (CDC P0) (Table 3).

**TABLE 3:** Mean percentage of cells [+Standard Deviation (SD)] expressing cell surface markers immediately post-digest (CDC P0) and in the separately cultured populations (CDC, NFAC and FAC) at Passage 1 (P1).

	CDC P0		CDC P1		NFAC P1		FAC P1	
	Mean	SD	Mean	SD	Mean	SD	Mean	SD
CD29	65.03	15.80	99.07	1.05	100.00	0.00	99.93	0.05
Cd44	6.05	3.28	97.33	2.10	95.20	4.37	88.40	15.06
CD56	0.28	0.01	32.60	45.68	17.49	24.62	0.67	0.60
CD73	97.13	0.66	100.00	0.00	100.00	0.00	99.97	0.05
CD90	21.07	5.55	97.50	2.01	97.57	1.44	84.60	12.92
CD49e	0.19	0.17	21.38	9.87	8.50	4.19	21.80	6.53
CD34	6.38	1.61	3.09	1.67	2.35	1.46	1.61	1.22
CD45	0.14	0.06	0.15	0.04	0.17	0.04	0.09	0.01
Stro-1	1.13	0.30	5.51	2.91	9.67	8.96	3.84	2.68
CD24	3.63	2.54	27.40	20.37	27.57	4.80	21.13	10.70

There were no significant differences between CDCs, NFACs or FACs for the expression of surface markers at P1 (Figure 2A). However, there were noted to be a greater proportion of cells expressing stem/progenitor cell markers (CD29, CD44, CD56, CD73, CD90, CD49e) and CD24 in the P1 populations compared to P0. Furthermore, these were statistically significant increases in cell percentage for CD29 (p < 0.01 for P0 vs. all P1 cells), CD44 (p < 0.0001 for P0 vs. all P1 cells) and CD90 (p < 0.0001 for P0 vs. all P1 cells) and a statistically significant reduction in cells positive for CD34 expression in the FAC and NFAC populations at P1 (p = 0.04). Notably, 100% of CDCs, NFACs and FACs expressed stem/progenitor cell marker CD73 at P1 and more than 99% expressed CD29. CD90 was also expressed in >97.5% of cells in the CDC and NFAC populations, and 84.6% in the FAC population. More CDC were CD56 positive at P1 (32.6%) compared to CDC at P0 (0.3%, p = 0.01) and to the FAC P1 population (0.7%, p = 0.01). There were no other significant differences noted between the P1 cell populations. Crucially, only 0.19% of CDCs expressed CD49e immediately after isolation, but this value increased to 8.5%–21.8% of all cell populations by P1.

No significant differences were observed between the three different cell populations at P1, indicating a drift to homogeneity in cell surface markers by first passage (Figures 2B, C; Supplementary Figure S2). Particularly stark was the increase in the CD49e signal at first passage from a mean S:N ratio of 1.26 to greater than 3 in the CDC and FAC populations (p = 0.004, p = 0.02 respectively). Similarly, there was a large rise in the S:N ratio of CD29 from a mean of 10.9 at P0 to 90 (CDC, p = 0.03), 209 (NFAC, p < 0.0001) and 154 (FAC, p < 0.0001) in the cell populations at P1.

There appeared to be significant differences in the presence of cell surface markers between P0 and P1, particularly increases in mesenchymal stem/progenitor cell markers such as CD29, CD44, CD73 and CD90. Taken together these data indicate that there are no significant differences between CDC, NFAC and FAC cell surface markers at P1 and that the emergence of mesenchymal stem/progenitor cell markers is likely an acquired phenotype as a product of cell culture conditions. The low number of cells expressing CD49e immediately post-isolation does not correlate with the number of cells capable of fibronectin adherence and the expression increases as a product of cell culture, making it an unreliable marker for confirmation of a chondroprogenitor cell line.

### 3.3 Histological evidence of trilineage differentiation

**FIGURE 3:**
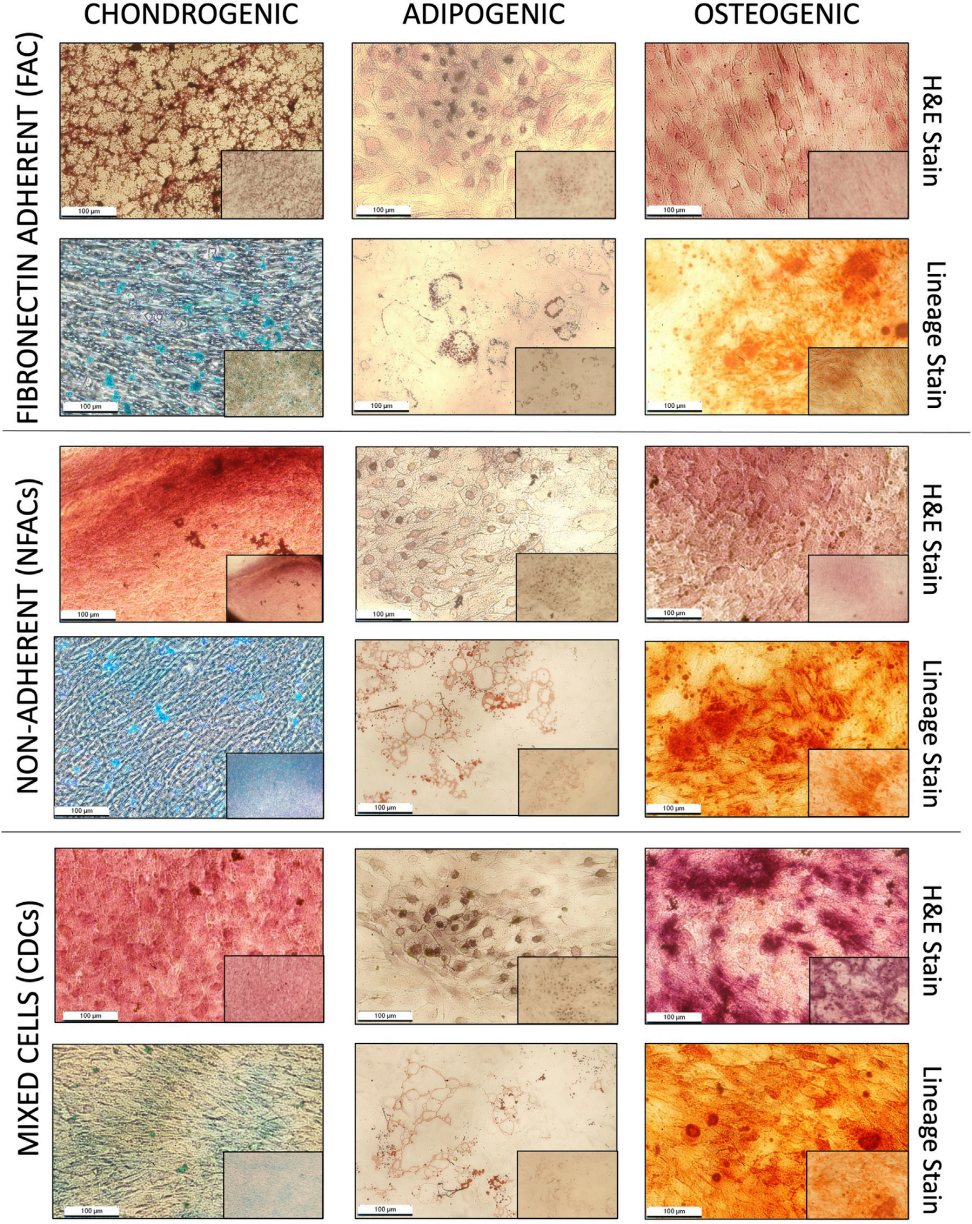
Histological analysis of P1 FACs, NFACs and CDCs directed down chondrogenic (left column), adipogenic (central column) and osteogenic (right column) lineages. All cells were stained for lineage specific stains (bottom row): Alcian blue for cartilage, Alizarin red for bone and Oil red O for adipose tissue in addition to H + E staining (top row) for cell morphology. All images presented depict cells taken at 20× magnification (large image) and 4× magnification (small image, bottom right).

To provide further confirmation of chondroprogenitor isolation, FACs are often demonstrated to undergo trilineage differentiation as a marker of a true mesenchymal stem/progenitor cell (Dominici et al., 2006). If the FACs are chondroprogenitors, they should be the only cell type capable of this phenomenon. It was noted here that as early as P1, all cell populations, not just those displaying fibronectin adherence, were capable of trilineage differentiation into osteocytes, chondrocytes and adipocytes, indicating multipotency. Staining with haematoxylin and eosin demonstrated cellular morphology consistent with a chondrocyte phenotype in all cell populations, confirmed with characteristic staining of glycosaminoglycan production with Alcian blue (Figure 3), also seen in the three chondrocyte populations without chondrogenic supplementation (Supplementary Figure S3). In the presence of osteogenic medium, all populations also demonstrated an ability to produce calcium deposits, as visualised with Alizarin red, consistent with successful osteogenic differentiation (Figure 3). Additionally, all cell types showed adipogenic differentiation, evidenced by large, round cells with cytoplasmic droplets on H&E staining, which when stained with Oil Red O stain, verified these as lipid droplets (Figure 3). As such, all cell types displayed an ability for trilineage differentiation in culture at P1 irrespective of fibronectin adherence, meaning trilineage differentiation is also an acquired ‘stem-like’ behaviour as a product of cell culture conditions and not exclusive to FACs.

### 3.4 Chondrogenic gene expression of nasoseptal cartilage cell populations

**FIGURE 4:**
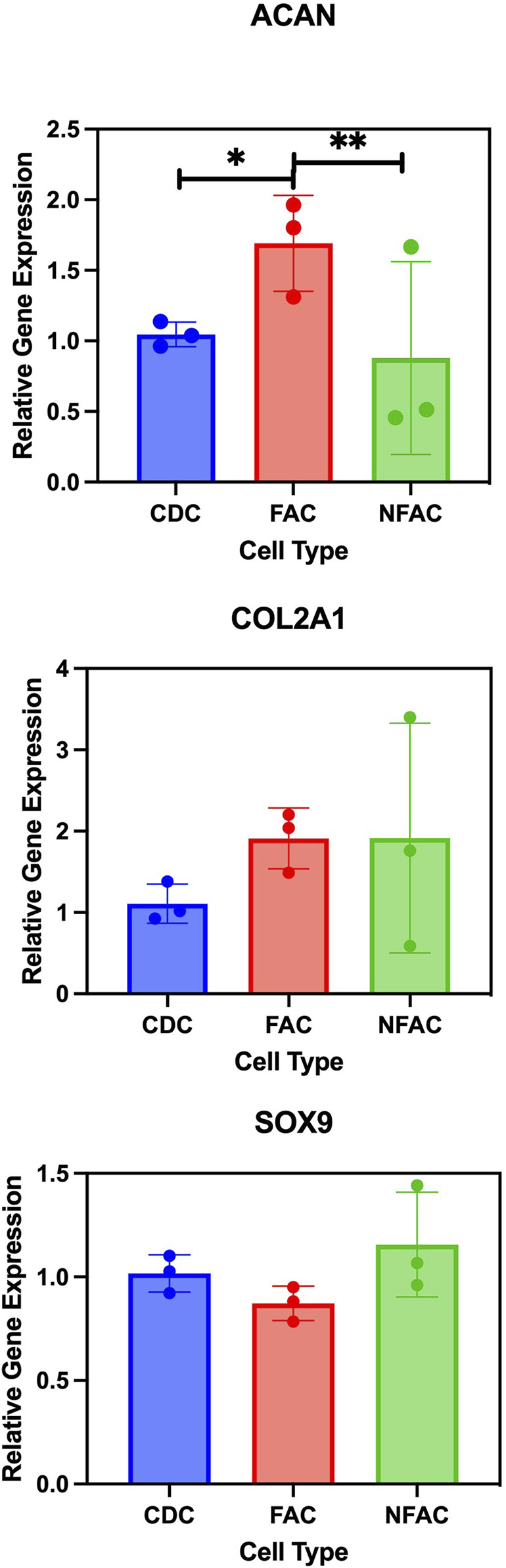
RT-qPCR analysis of chondrogenic gene markers Type 2 Collagen (COL2A1); Aggrecan (ACAN) and SOX9. Mean FAC and NFAC gene expression is reported relative to CDC at P1 from 3 biological repeats (in technical triplicates). Statistical comparisons made using nested ANOVA analysis with Tukey’s *post hoc* test are displayed with error bars depicting standard deviation. * = p < 0.05, ** = p < 0.01.

Whilst phenotypically similar and all demonstrating trilineage differentiation, there may be biological differences in the CDC, FAC and NFAC populations that convey superior characteristics of significance for cartilage tissue engineering such as chondrogenic gene expression. To characterise chondrogenic potential, cell populations were next examined for differences at the level of chondrogenic gene expression. P1 CDCs, FACs, and NFACs were harvested and the baseline gene expression of chondrogenic markers SOX9, COL2A1 and ACAN determined relative to housekeeping genes TBP and RPL13A (Figure 4).

There were no significant differences in gene expression between the FAC, NFAC and CDC populations for SOX9 expression. However, there was a significant difference in the expression of aggrecan between the cell populations: the FAC population had significantly higher ACAN expression than CDCs (1.7-fold increase, p = 0.0082) whereas no difference was observed between the CDC and NFAC population (p = 0.72). When comparing COL2A1 expression across the three cell populations, there were differences between the CDC population and FAC population (2.0-fold higher; p = 0.012) but not the CDC and NFAC populations (1.5-fold higher, p = 0.29). In summary, at P1, the FAC population appears to possess superior extracellular matrix gene expression (ACAN and COL2A1) compared to the CDC population, whilst the NFAC population was comparable in all gene expression to the CDC population.

Therefore, fibronectin adherence is associated with greater expression of extracellular matrix genes: ACAN and COL2A1, however it is not possible to determine whether this phenomenon is a characteristic associated with cells that preferentially adhere to fibronectin or whether the adherence to fibronectin has evoked this change in gene expression.

### 3.5 Adherence and proliferation of nasoseptal cartilage populations

**FIGURE 5:**
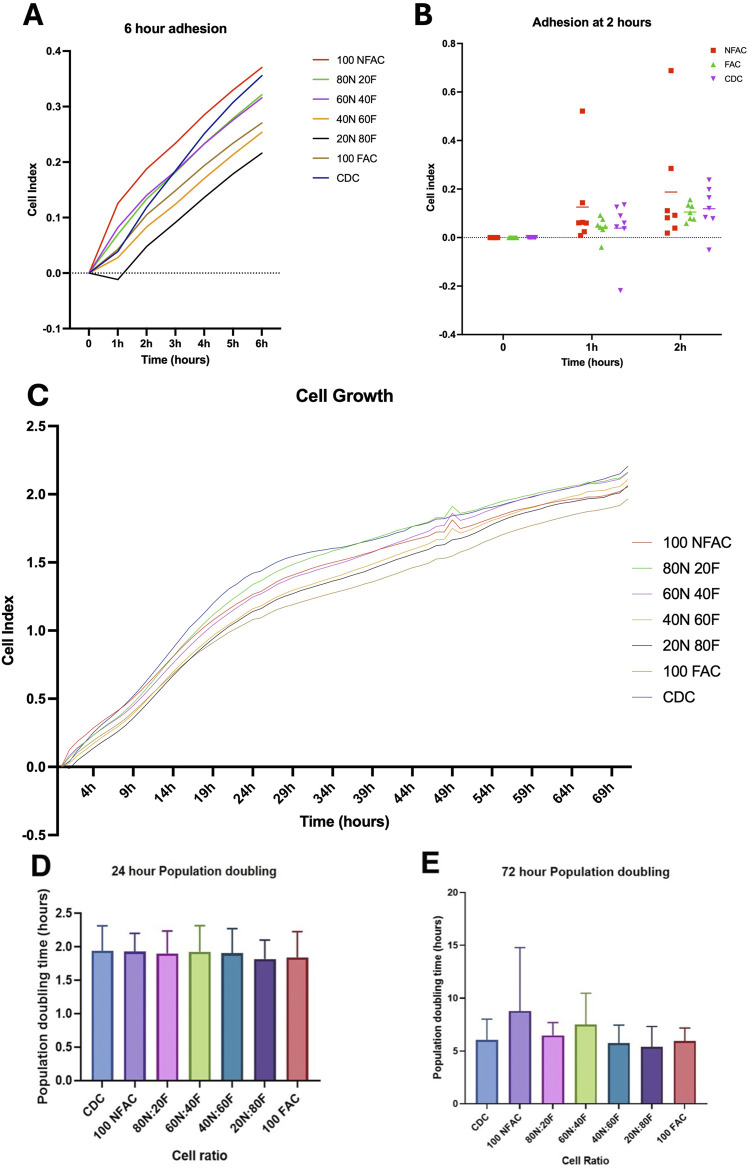
Adhesion and proliferation of nasoseptal cartilage cells over 72-h time course. Mean values of 7 biological repeats performed in technical duplicates with standard deviation are shown. **(A)** Adhesion rates in different cell populations at 0-, 1- and 2-h time points compared to media. Statistical significance was observed at 2 h in the CDC and FAC populations. **(B)** Cells of differing ratios of FAC and NFAC were compared to CDC populations and media only controls. Time is plotted on the *x*-axis against cell index on the *y*-axis. Significance at the 6-h timepoint is demonstrated compared to media. **(C)** iCELLigence-acquired cell growth data over a 72-h time period for the different ratios of cell populations compared to media only controls (blue). No statistically significant differences were observed between cell populations. **(D, E)** Mean population doubling times taken for the first 24 h of culture **(D)** using the iCELLigence device and for the entire experimental period **(E)** of 72 h. No statistically significant differences were seen between cell populations. * = p < 0.05, ** = p < 0.01, **** = p < 0.0001.

Cells must also demonstrate plastic adherence to be considered as mesenchymal stem/progenitor cells (Dominici et al., 2006). In order to assess this characteristic, we sought to confirm that all cell populations examined exhibited this feature using an iCELLigence impedance-based approach apparatus to characterise cellular plastic adherence and growth characteristics over a 72-h period (Figure 5A). This technology has been previously validated for measuring adhesion and proliferation (Jessen et al., 2022; Zhao et al., 2024) There were no statistically significant differences between cell populations in terms of adherence at 0 or 1 h post seeding (Figure 5A). Statistically significant changes were seen in the ANOVA model across the three time points indicating progressive adherence (p < 0.0001) with the FAC and CDC cell population demonstrating statistically significant cell adhesion had occurred (CDC p = 0.007; NFAC p = 0.4; FAC p = 0.03).

Different proportions of NFACs and FACs were then combined to determine the effect on adhesion and growth kinetics and to determine whether superior combinations of cells could be produced for cartilage tissue engineering. All cell ratio populations were found to have evoked statistically significant changes in electrical impedance by 6 h (Figure 5B) with mean cell indices of 0.46 (100NFAC, p = 0.02), 0.41 (80N20F, p = 0.02), 0.40 (60N40F, p = 0.04), 0.34 (40N60F, p = 0.0002), 0.30 (20N80F, p = 0.006), 0.35 (100FAC, p = 0.02) and 0.44 (CDC, p = 0.007). However, at 6 h there were no statistically significant differences between any of the different cell ratio populations. Therefore, all cells demonstrated an ability to adhere to plastic, but this appeared to occur more readily in the CDC and FAC populations.

To assess for disparities in growth kinetics, cell growth was measured in the different cell populations using iCELLigence over the course of 72 h (Figure 5C). A two-way repeated measures ANOVA indicated that time (p < 0.0001), cell population (p < 0.0001) and patient (p < 0.0001) were all statistically significant sources of variation, however, no significant differences were observed between cell populations. A more rapid increase in cell index was observed over 24 h than 72 h (Figures 5D, E) however these were not significantly different between any cell populations (p > 0.99).

### 3.6 Tissue engineering potential of different nasoseptal cell populations

**FIGURE 6:**
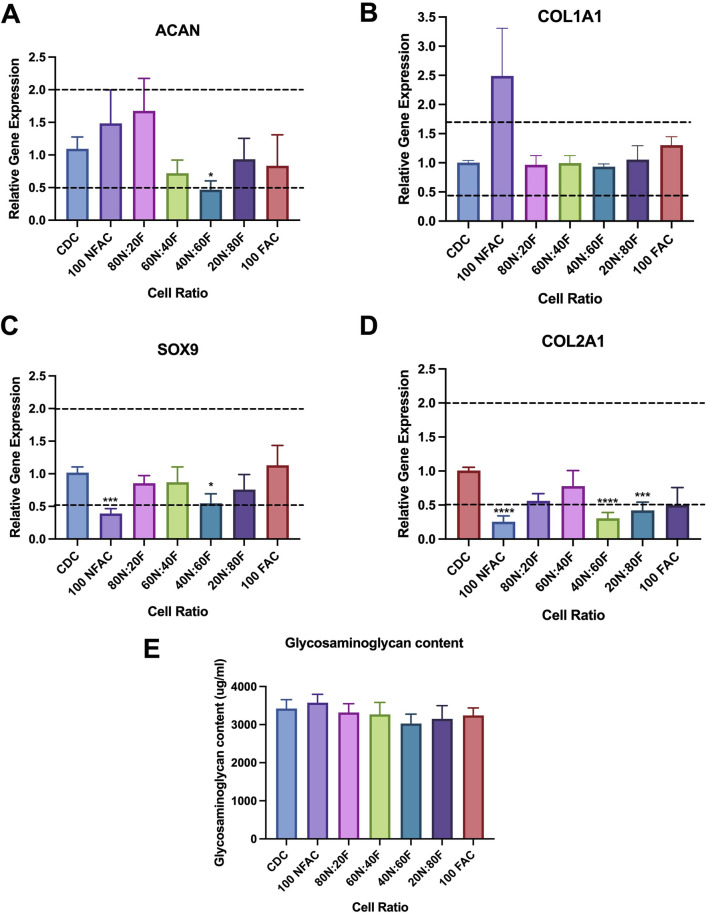
Chondrogenic potential of nasoseptal cartilage cell populations after 21 days of coculture in alginate beads. For brevity, NFAC has been shortened to N and FAC to F **(A–D)** Mean gene expression of chondrogenic markers relative to CDC population. Horizontal lines at 0.5 and 2 indicate biological significance thresholds. **(A)** ACAN gene expression, no significant difference seen relative to CDC except for reduced expression in 40N:60F. **(B)** COL1A1 expression, no significant differences seen in any cell groups **(C)** SOX9 expression, significantly lower gene expression was noted in 100NFAC and 40N:60F only. **(D)** COL2A1 gene expression, significantly lower gene expression was seen in 100N; 40N:60F and 20N:80F populations. **(E)** DMMB Assay demonstrating glycosaminoglycan content of cell populations cultured in alginate after 21 days, in which no significant differences were seen. All bars represent mean values with standard deviation. * = p < 0.05; *** = p < 0.001; **** = p < 0.0001.

There is evidence to suggest that 2D tissue culture models may evoke chondrocyte dedifferentiation: a phenomenon that can be reversed by culturing in a 3D environment such as within an alginate bead (Homicz et al., 2002; Caron et al., 2012; Aurich et al., 2018). As such, the next stages of this experiment explored the chondrogenic potential of the diffferent cell populations in 3D culture to identify whether a superior combination of cell types could be exploited for tissue engineering applications. To determine whether FAC and NFAC populations conferred any beneficial effects for cartilage tissue engineering in a 3D hydrogel environment, different ratios of NFAC and FAC cells were cultured in 3D alginate beads and gene expression relative to the CDC population was analysed at 21 days (Figures 6A–D). Multiple comparisons were conducted using a one-way Kruskal–Wallis ANOVA against the CDC population as a control, to which the relative gene expression was calculated. SOX9 expression was largely uniform across cell populations, but with significantly lower expression in the 100NFAC (0.39-fold difference, p = 0.0001) and 40N:60F populations (0.55-fold difference, p = 0.013). ACAN similarly demonstrated minimal signficant difference between cell populations, but a signifcant reduction of 0.47-fold was noted in the 40N:60F cell mixture in alginate (p = 0.02). Whilst a higher mean relative expression of COL1A1 was noted in the NFAC population (2.49-fold difference) this was not signifcant (p = 0.3). Whereas COL2A1 was the gene in which the largest variation was seen, with many populations having lower gene expression than CDCs: 100NFAC (0.25-fold, p < 0.0001), 40N:60F (0.31-fold, p < 0.0001), 20N:80F (0.42-fold, p = 0.0005). Therefore, no combinations of NFAC and FAC cells conferred superior chondrogenic gene expression relative to CDCs, meaning isolation of cells on the basis of fibronectin adherence is unlikley to be of benefit for cartialge tissue engineering.

**FIGURE 7:**
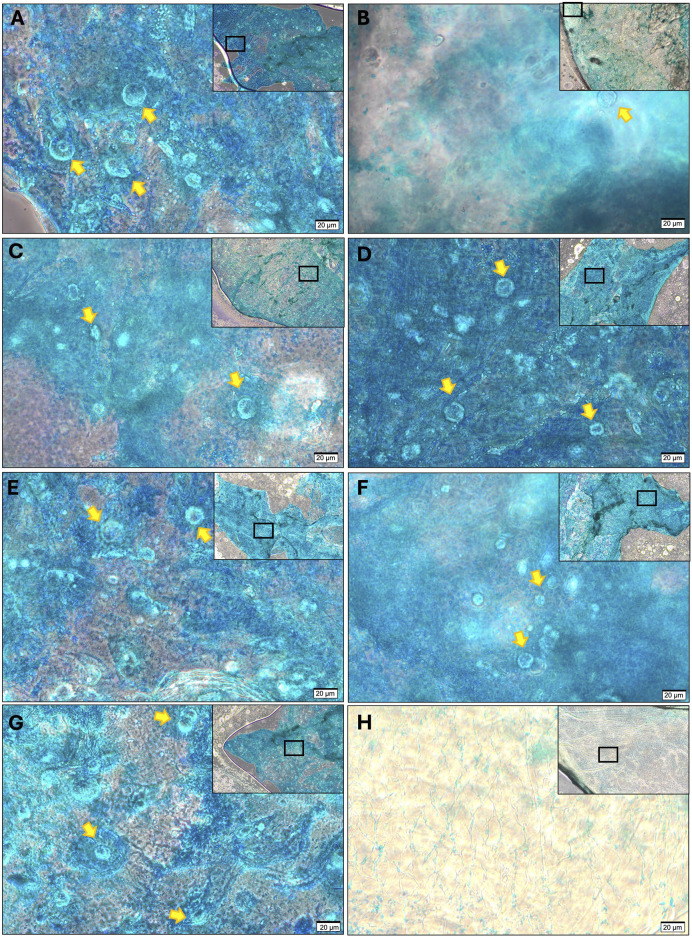
Histological analysis of GAG production from cell ratio populations in alginate culture at 21 days stained with Alcian Blue. **(A)** CDC, **(B)** 100N, **(C)** 80N:20F, **(D)** 60N:40F, **(E)** 40N:20F, **(F)** 20N:80F, **(G)** 100F; compared to **(H)** acellular control stained with alcian blue. All sections demonstrate the presence of chondrocytes (highlighted with yellow arrows) and a peripheral rim of intense alcian blue staining except for **(B)** where weak correlation between stain and cells was observed. All images were acquired at 40× magnification with scale bars denoting 20 μm, lower magnificent (10×) images are displayed in the top right of each image, with rectangular frames to show the area from where the representative 40× image was taken.

Differences in gene expression did not however translate to differences in the amount of extracellular matrix produced (Figure 6E). Over 3 mg/mL of glycosaminoglycan was detected in all cell ratio protein lysates however no statistically significant differences were observed between mixed cell populations after 21 days of culture. Histologically (Figure 7), there was evidence of glycosaminoglycan deposition in a pericellular location in most of the cell populations in 3D alginate culture, validating the findings of the DMMB assay. The pericellular deposition in the 100NFAC population was less convincing that the other cell populations however in the studied sections. This data indicates that FACs and NFACs have similar chondrogenic profiles in long term 3D culture, but moreover that they have no benefit compared to CDCs alone for enhancing extracellular matrix production for cartilage tissue engineering.

## 4 Discussion

This study aimed to identify the most suitable cells, or combination of cells, from nasoseptal cartilage tissue for tissue engineering purposes. In order to address this aim, we determined the native proportions of fibronectin adherent (presumed chondroprogenitor cells) and non-adherent cells in both male and female patients, assessed the validity of the fibronectin adhesion assay in isolating a population of cells demonstrating mesenchymal stem/progenitor cell properties, and explored whether different combinations of FACs and NFACs could be generated to surpass the chondrogenic potential of native cell mixtures (CDCs).

Cartilage tissue is notorious for its poor ability to renew and regenerate, underpinning cartilage specific pathologies such as osteoarthritis (Hunziker et al., 2015). As such, attention has been focussed towards isolating a population of stem/progenitor cells within cartilage tissue, which could be manipulated to facilitate repair and regeneration (Williams et al., 2010a). Whilst there has been significant interest in isolating and characterising chondroprogenitor cells from articular cartilage, fewer have focussed on identifying and characterising chondroprogenitors from nasoseptal cartilage (Jessop et al., 2019; Jessop et al., 2020). Nonetheless, the use of the fibronectin adhesion assay to isolate chondroprogenitors on the basis of CD49e expression remains a dominant technique in the field of cartilage biology (Jessop et al., 2019; Jessop et al., 2020; Kachroo et al., 2020).

When first described in articular cartilage in 2010, 0.7% of the total cells analysed demonstrated CD49e positivity, and possessed the ability to clonally proliferate, expressed Notch1, retained stem/progenitor cell surface markers CD90 and Stro-1 and were capable of trilineage differentiation (Williams et al., 2010b). Other studies of articular and auricular cartilage since have indicated that values as high as 2%–3% of the total cells may be chondroprogenitor cells using a range of isolation methods including clonal expansion and cell sorting, in addition to fibronectin adherence (Jessop et al., 2019). Here, we have shown that there are a proportion of cells that preferentially adhere to fibronectin within 20 min of coculture: FACs, which should map to the CD49e positive cells as reported previously. Indeed, the FAC population does appear to meet many of the mesenchymal stem/progenitor cell characteristics defined by Dominici et al. (2006): expressing key stem/progenitor cell markers such as CD73 and CD90, but lacking the expression of CD45 and CD34, demonstrating an affinity for plastic adherence, and having a capacity for trilineage differentiation. However, the FAC population in both male and female subjects comprised over 50% of the cells isolated from nasoseptal cartilage, thereby being the dominant cell population in nasoseptal cartilage. However, only 0.19% of all CDCs liberated from nasoseptal cartilage were found to be CD49e positive prior to commencing culture (P0): a value that increased to greater than 21% of cells by P1 in the CDC and FAC populations.

These findings indicate that the cell surface expression of CD49e alone is therefore unlikely to be solely accountable for fibronectin adherence. Indeed recent studies of articular cartilage have described a similar phenotypic drift in cultured chondrocytes, with increases in CD49e expression noted as early as 24 h in culture (Kachroo et al., 2020). The overt disparity in CD49e expression (0.19%) and fibronectin adherence (>50%) indicates another cell surface marker must be facilitating fibronectin adherence. The fibronectin receptor, also known as the α5β1 integrin receptor, is a heterodimeric membrane protein, in which CD49e comprises the integrin α5 chain. The β1 subunit with which α5 (CD49e) dimerises, is also known as CD29, and in this study, the expression of CD29 in CDCs at P0 is approximately 65%. This more closely aligns to the numbers of cells adhering to fibronectin than CD49e, which indicates that the β1 subunit may be expressed on CDCs with an alternative alpha subunit capable of fibronectin adherence. Integrin β1 is one of the most widely expressed beta integrins but associates with at least 10 different alpha integrins (Hynes, 1992), of which many are capable of fibronectin adhesion (Elsaesser et al., 2016; Zhang et al., 1993). As such, this assay lacks the specificity to isolate cells purely on the basis of CD49e expression.

The phenotypic drift observed in this study extends beyond the scope of CD49e expression. FACs were able to demonstrate the main International Society of Cellular Therapy criteria in being plastic adherent, expressing key mesenchymal stem/progenitor cell markers and being capable of trilineage differentiation (Dominici et al., 2006), yet so too were CDC and NFAC cell populations at P1. Moreover, all cells demonstrated an ability to differentiate into osteogenic, chondrogenic and adipogenic lineages, suggestive of multipotent stem/progenitor cell characteristics. This finding mirrors recent studies of articular chondrocytes, and calls for a more reliable set of markers of chondroprogenitor cells to be identified (Vinod et al., 2020). Upon immediate isolation of CDCs from nasoseptal cartilage, the expression of key stem/progenitor cell markers was relatively low with 6% of cells expressing CD44, 21% expressing CD90 yet 97% expressing CD73. However by P1, CD44, CD73, CD90 and CD29 were expressed on almost all cells regardless of their ability to adhere to fibronectin. Of note, many of these markers (CD44, CD29 and CD90) are all involved in mediating cell-matrix and cell-cell adhesion. Whilst it has been demonstrated that CD49e and CD29 expression has been shown to increase within 24 h in culture conditions, we demonstrate that the phenotypic drift is even more stark, with all cells possessing multipotent stem/progenitor cell-like surface markers and behaviours as early as P1. Whilst, this has been previously attributed to culturing cells in plastic monolayer conditions (Homicz et al., 2002; Darling and Athanasiou, 2005), more recent studies have identified this phenomenon also occurs in non-adherent cell cultures (Kachroo et al., 2020). Whether it is the conditions of cell culture or the separation of chondrocytes from their resident extracellular matrix that evokes this change remains uncertain, but the phenotypic drift of all cartilage cells post-extraction is problematic for tissue engineering purposes. The significant heterogeneity in published stem/progenitor cell surface markers, reiterates a need to clarify and refine a reliable set of cell surface markers to distinguish progenitor cells from chondrocytes and dedifferentiated cells in culture. Owing to the plasticity of chondrocytes post-isolation, the International Society of Cellular Therapy criteria appear to lack the specificity to delineate chondroprogenitors from dedifferentiated chondrocytes which may confound efforts to isolate true progenitor cell populations from cartilage (Dominici et al., 2006). A limitation of this study is that there was variability in between the biological repeats for certain cell surface markers (particularly CD56, CD49e and CD24) which may have masked further significant differences between cells and timepoints.

Whilst cell surface markers and trilineage differentiation are important considerations for cell selection in cartilage tissue engineering, if fibronectin adherent cells possess superior chondrogenicity or proliferation characteristics, their value in tissue engineering should not be overlooked. In previous studies, FACs from nasoseptal cartilage were found to express higher levels of chondrogenic genes, such as type 2 collagen (Jessop et al., 2020). Within native cartilage tissue, it would be expected that chondrocytes, rather than chondroprogenitors would express higher levels of extracellular matrix genes such as type 2 collagen and aggrecan, owing to their physiological role in maintaining the ECM (Lefebvre et al., 2019). However, as in the previous study, the inverse was observed in our study: the expression of these genes was higher in the FAC population. Fibronectin is only present in small amounts in cartilage tissue but is believed to have a role in organising the extracellular matrix and has been implicated in promoting chondrogenic differentiation (Casanova et al., 2020). We hypothesise that the increase in aggrecan and COL2A1 gene expression may actually be a product of exposure to fibronectin, rather than an inherent phenotype of FACs (Singh and Schwarzbauer, 2014; Casanova et al., 2020).

In order to circumvent previous reports of chondrocyte dedifferentiation in 2D culture (Diaz-Romero et al., 2005; 2008; Grogan et al., 2007; Hamada et al., 2013), we attempted to culture cells in a 3D alginate bead, firstly to mirror the conditions of tissue engineering, but also as this has previously been reported as sufficient to redifferentiate chondrocytes into their native phenotypes (Caron et al., 2012; Aurich et al., 2018; Kisiday, 2019). It was apparent that there were no overtly superior ratios of FAC and NFAC in 3D culture relative to CDC populations in this study, the latter of which consistently demonstrated superior chondrogenic gene expression. Similarly, there were no significant differences observed in the growth kinetics or level of extracellular matrix production in any cell combination studied. These findings mirror a recent study of articular cartilage chondroprogenitor cells combined with articular chondrocytes in ratios of 80:20, 65:35, 50:50, 35:65 and 20:80 at s passage (Vinod et al., 2019). There was similarly no difference in population doubling times, cell surface markers, or chondrogenic gene expression (COL2A1, ACAN and SOX9) between any of these articular cell mixes (Vinod et al., 2019), validating the findings in our study of nasoseptal chondrocytes.

As 3D culture conditions should correct for dedifferentiation, yet demonstrate homogeneity in the glycosaminoglycan content and gene expression profiles in the different cell ratios, we conclude that there are no convincing differences in the behaviours of the cell populations separated by fibronectin adherence. Previous studies have used mesenchymal stem/progenitor cells derived from adipose tissue, bone marrow and synovium combined with chondrocytes to successfully enhance chondrogenesis (Chang et al., 2011; Kubosch et al., 2016; Arora et al., 2017). This phenomenon did not appear to be replicated in this instance with fibronectin adherent cells, casting further doubt on their synonymity with chondroprogenitor cells.

## 5 Conclusion

In this study, we have demonstrated firstly, that fibronectin adhesion does not specifically isolate cells on the basis of CD49e expression; secondly, that irrespective of fibronectin adherence, cultured nasoseptal cartilage cells demonstrate a rapid phenotypic drift in which they emulate mesenchymal stem/progenitor cells in both phenotype and behaviour, and thirdly, that cells separated on the basis of fibronectin adherence confer no advantages for cartilage tissue engineering purposes.

These findings, combined with similar findings in the field of articular chondroprogenitor isolation, indicate that the fibronectin adhesion assay is unreliable for chondroprogenitor isolation, and that additional research into identifying, characterising and isolating chondroprogenitor cells is warranted if the intention is to exploit the limited regenerative capacity of cartilage for tissue engineering and regenerative medicine applications. The application of transcriptomics and next-generation sequencing technologies has provided an alternative means of assessing differences between articular cartilage cell populations (Dicks et al., 2020), and may offer deeper insight into differentiating features of nasoseptal chondroprogenitor and chondrocyte populations in future research.

## Data Availability

The raw data supporting the conclusions of this article will be made available by the authors, without undue reservation.
