# Supplementary material for: Nasoseptal chondroprogenitors isolated through fibronectin-adherence confer no biological advantage for cartilage tissue engineering compared to nasoseptal chondrocytes
Source: Front Bioeng Biotechnol. 2024 Sep 26;12:1421111. doi: 10.3389/fbioe.2024.1421111 (PMC11464323; doi:10.3389/fbioe.2024.1421111)
Supplement: Supplementary file 1 [file DataSheet1.docx]

**Supplementary Figures and Tables**

*Supplementary Table 1:* Primer sequences used for qPCR analysis

| Gene |  | Primer sequence | Accession number | Product size (base pairs) |
| --- | --- | --- | --- | --- |
| TBP | Forward  Reverse | 5’-GCCAAGAGTGAAGAACAG  5’-GAAGTCCAAGAACTTAGCTG | NM_001172085.2 | 90 |
| RPL13A | Forward  Reverse | 5’-GTCTGAAGCCTACAAGAAAG  5’-TGTCAATTTTCTTCTCCACG | NM_012423.4 | 189 |
| ACAN | Forward  Reverse | 5’-CACCCCATGCAATTTGAG  5’-AGATCATCACCACACAGTC | NM_001135.4 | 82 |
| COL2A1 | Forward  Reverse | 5’-GAAGAGTGGAGACTACTGG  5’-CAGATGTGTTTCTTCTCCTT | NM_001844.5 | 165 |
| SOX9 | Forward  Reverse | 5’-CTCTGGAGACTTCTGAACG  5’-AGATGTGCGTCTGCTC | NM_000346.4 | 172 |


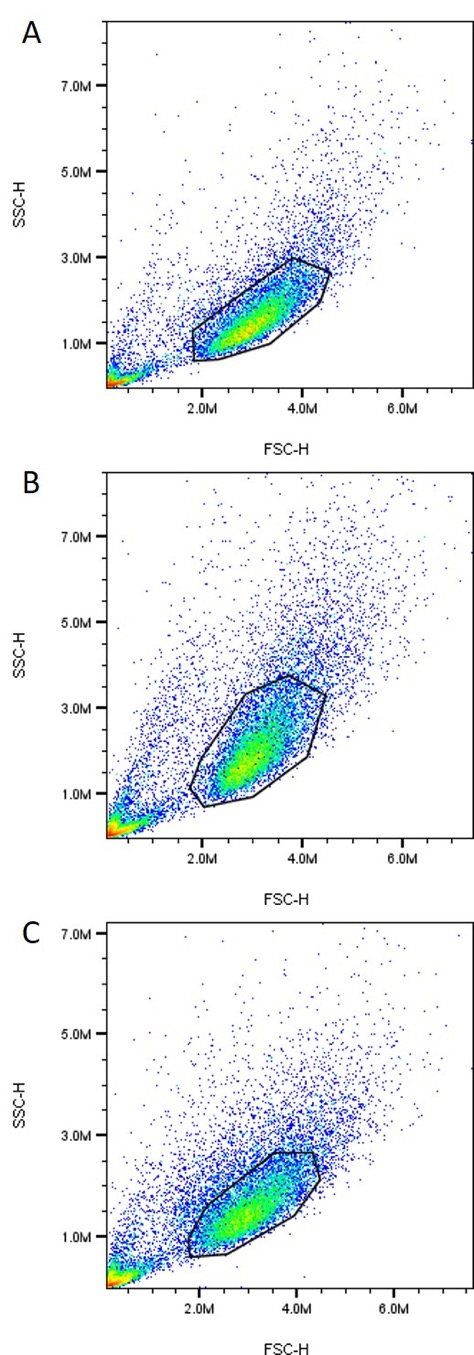
Supplementary Figure 1: Flow cytometry scattergrams plotted using forward (FSC-H) and side scatter (SSC-H) for identifying and gating cell populations. CDC populations (A), NFAC populations (B) and FAC populations (C) were each gated for the most homogeneous, viable cell population as drawn. The 100,000 cells gated in each sample were used in subsequent analysis for cell surface markers. Representative images are shown from a single biological sample for illustrative purposes.


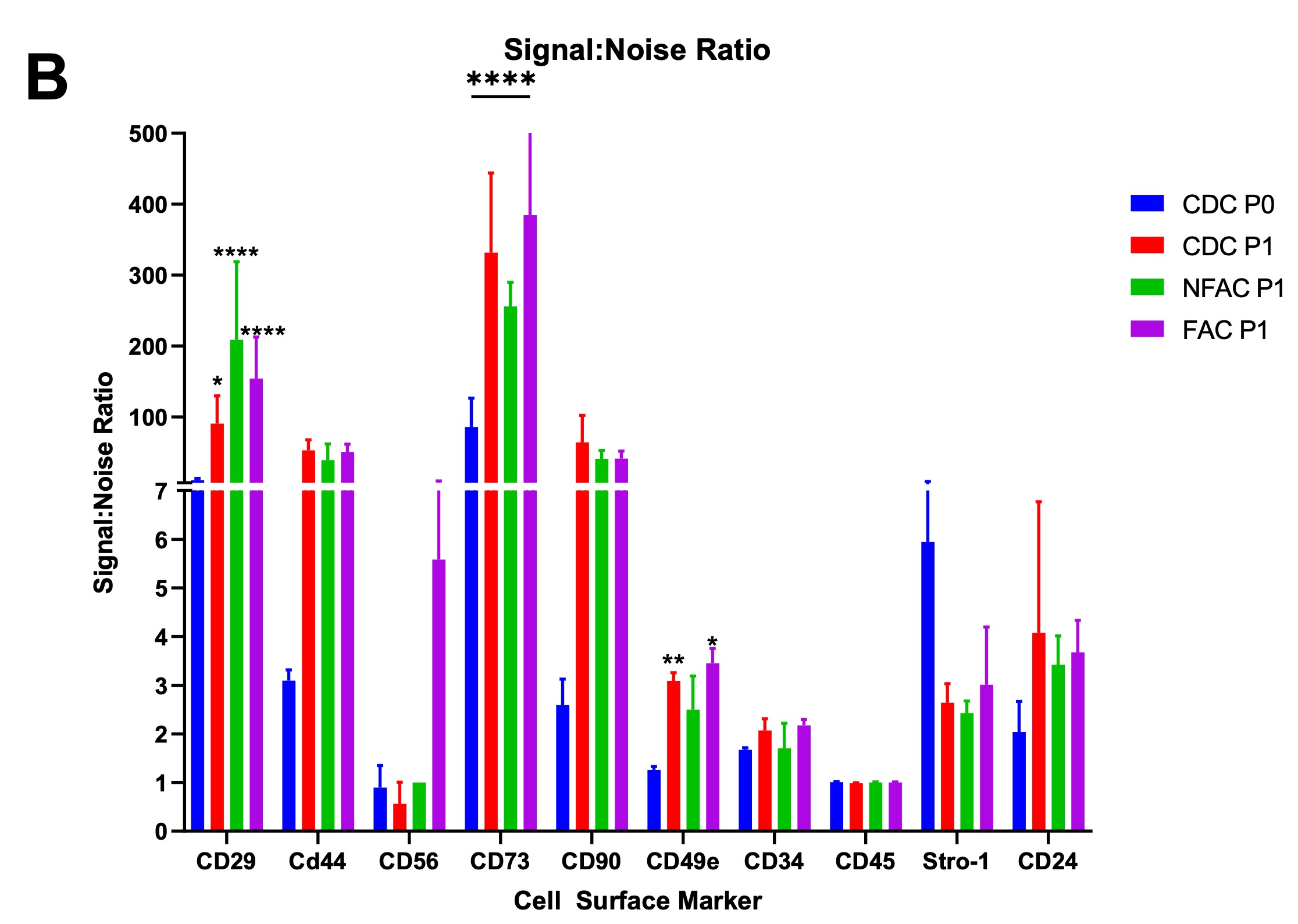


*Supplementary Figure 2:* Signal:Noise ratios derived from median fluorescence index (MFI) of stained populations corrected for the MFI of the unstained population. A S:N ratio of 1 is therefore equivalent to no fluoresence. Each cell surface marker is examined in the full cell population immediately post-digest at P0 (blue, CDC only) and then in the three populations at P1 (CDC = red, NFAC=green, FAC=purple). The mean S:N ratio of three biological replicates are presented with SD error bars. *=p<0.05; **=p<0.01; ***=p<0.001; ****=p<0.0001.


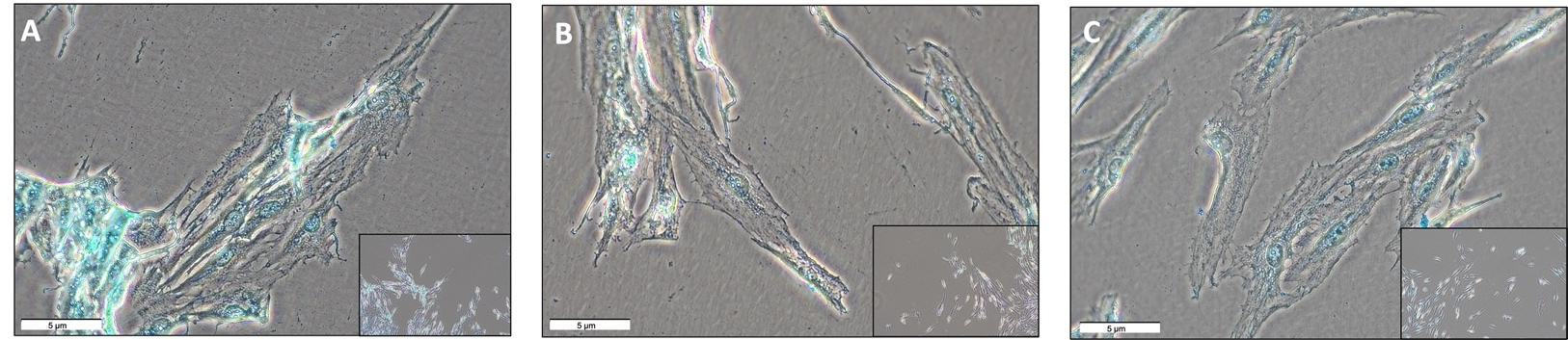


*Supplementary Figure 3:* Chondrocyte populations stained with Alcian Blue stain at Passage 1. A) CDCs B) NFACs C) FACs are demonstrated at 40x magnification with 10x magnification in the bottom right corner.
